# Echinacoside Induces Apoptosis in Human SW480 Colorectal Cancer Cells by Induction of Oxidative DNA Damages

**DOI:** 10.3390/ijms160714655

**Published:** 2015-06-29

**Authors:** Liwei Dong, Debin Yu, Nuoting Wu, Hongge Wang, Jiajing Niu, Ye Wang, Zhihua Zou

**Affiliations:** Key Laboratory for Molecular Enzymology and Engineering of the Ministry of Education, National Engineering Laboratory for AIDS Vaccine, School of Life Sciences, Jilin University, 2699 Qianjin Street, Changchun 130012, China; E-Mails: donglw10@mails.jlu.edu.cn (L.D.); yudebin4685_cn@163.com (D.Y.); wunuoting@163.com (N.W.); wanghg13@mails.jlu.edu.cn (H.W.); niujj14@mails.jlu.edu.cn (J.N.); wangye0106@jlu.edu.cn (Y.W.)

**Keywords:** Echinacoside, apoptosis, cell cycle arrest, DNA damage, 8-oxoG

## Abstract

Echinacoside is a natural compound with potent reactive oxygen species (ROS)-scavenging and anti-oxidative bioactivities, which protect cells from oxidative damages. As cancer cells are often under intense oxidative stress, we therefore tested if Echinacoside treatment would promote cancer development. Surprisingly, we found that Echinacoside significantly inhibited the growth and proliferation of a panel of cancer cell lines. Treatment of the human SW480 cancer cells with Echinacoside resulted in marked apoptosis and cell cycle arrest, together with a significant increase in active caspase 3 and cleaved PARP, and upregulation of the G1/S-CDK blocker CDKN1B (p21). Interestingly, immunocytochemistry examination of drug-treated cancer cells revealed that Echinacoside caused a significant increase of intracellular oxidized guanine, 8-oxoG, and dramatic upregulation of the double-strand DNA break (DSB)-binding protein 53BP1, suggesting that Echinacoside induced cell cycle arrest and apoptosis in SW480 cancer cells via induction of oxidative DNA damages. These results establish Echinacoside as a novel chemical scaffold for development of anticancer drugs.

## 1. Introduction

Echinacoside is a natural compound derived from the medicinal plant species of the genus of *Cistanche* [1,2] as well as *Echinacea* [3,4]. Extracts from the stem of different parasitic *Cistanche* plants have been used as a tonic in China for thousands of years [2], and extracts of *Echinacea* is one of the most popular herbal supplements in the US and Europe [3], being used widely to treat common cold and infections [4]. However, these herbal products are commonly used without the supervision of a healthcare provider; the efficacy, especially the safety of their use, have not been rigorously tested and confirmed.

**Figure 1:**
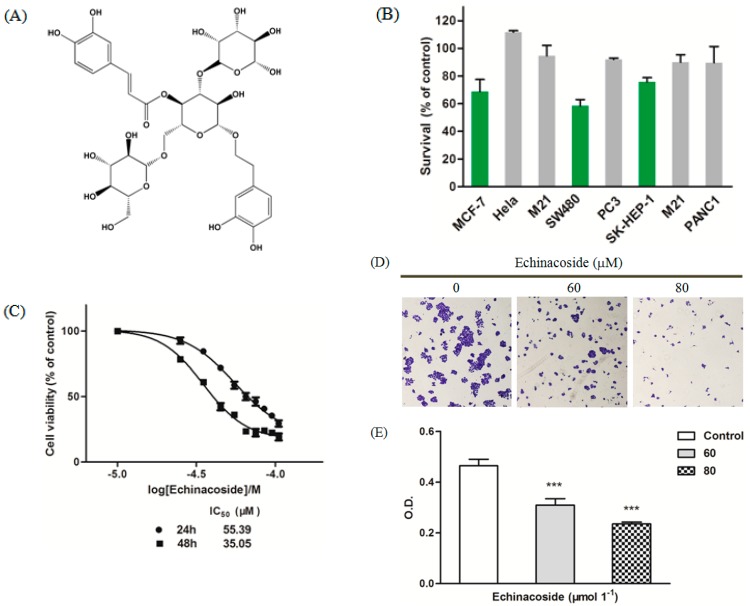
(**A**) Chemical structure of Echinacoside; (**B**) MTT assay: Cells were treated with 50 μM Echinacoside for 24 and cell viability was determined by MTT assay; (**C**) Dose-dependent inhibition curve in SW480 cells: Cells were treated with 25, 35, 45, 55, 65, 75, 85 and 95 μM Echinacoside for 24 and 48 h and cell viability was determined by MTT assay, data were analyzed by the GraphPad Prism software; (**D**) Images of colony formation assay: Cells were treated with 0, 60 and 80 μM Echinacoside for 10 days and stained with crystal violet; and (**E**) Quantification of colony formation assay: Crystal violet-stained cells were dissolved in 70% ethanol, and absorbance at 595 nm was measured by a microplate reader (*** *p* < 0.001 *vs*. vehicle control).

Echinacoside is one of the major bioactive herbal ingredients isolated from *Cistanche* and *Echinacea* plants [1]. It is a hydrophilic polyphenol glycoside consisting of a phenylpropanoid and a phenylethanoid glycosidically linked to a trisaccharide moiety (Figure 1A). Many biological effects have been ascribed to Echinacoside, including neuroprotection [5], hepatoprotection [6], anti-apoptosis [7], anti-senescence [8], immunomodulation [9], aperient [10], anti-diabetes [11] and promotion of bone formation [12], but detailed molecular mechanisms behind these effects remain unclear. Perhaps the most extensively studied and accepted bioactivity of Echinacoside is its anti-oxidative and reactive oxygen species (ROS)-scavenging function [13,14], which may also explain its neuroprotective and anti-aging effects.

ROS oxidizes lipids, proteins, nucleic acids, as well as free nucleotides in the cellular and mitochondrial dNTP pools [15]. Oxidized dNTPs are important sources of oxidative DNA damages [16], which may lead to cellular senescence and apoptosis, implicating ROS in aging and aging-related diseases [17]. Thus, preventing dNTP oxidization by antioxidants is believed to be beneficial to the overall health [18]. However, cancer cells generate much higher ROS and are highly dependent on efficient prevention of ROS-associated DNA damages for survival [19]. In this regard, highly proliferating cancer cells may benefit more from the anti-oxidative and ROS-scavenging activities of antioxidants than normal cells. In fact, several recent studies have shown that antioxidants promote the development of some forms of cancer [20,21,22,23].

Very few studies have examined the effects of Echinacoside on different types of cancer cell. Here, we treated a panel of cancer cell lines with Echinacoside to see if its anti-oxidative function would promote cancer cell survival. Surprisingly, we found that Echinacoside significantly inhibited the growth and proliferation of SW480 cancer cells by induction of cell cycle arrest and apoptosis. Given its many other health beneficial bioactivities, these results establish Echinacoside as a novel chemical scaffold for the development of anticancer drugs.

## 2. Results and Discussion

### 2.1. Echinacoside Blocked Proliferation of SW480 Cells

To investigate the effect of Echinacoside on cancer cells, we treated a variety of human cancer cell lines with 50 μM Echinacoside. MTT assay showed that, instead of being protective, Echinacoside inhibited the growth of human SK-HEP-1 hepatoma, MCF-7 breast cancer and SW480 colorectal cancer cells, with SW480 cells being the most sensitive (Figure 1B). We then focused our studies on SW480 cells. Treatment of SW480 cells with different doses of Echinacoside showed that Echinacoside dose-dependently inhibited the growth of SW480 cells (Figure 1C). The IC_50_ of 24 and 48 h treatment were 55.39 and 35.05 μM respectively (Figure 1C).

Next, we confirmed the effects of Echinacoside on SW480 cells by colony formation assay (Figure 1D,E). After treatment with 60 and 80 μM Echinacoside, SW480 cells formed fewer colonies, and the colonies that formed were much smaller (Figure 1D). Together, these results showed that Echinacside dose-dependently inhibited the proliferation of SW480 cells (Figure 1E).

### 2.2. Echinacoside Arrested SW480 Cells at G1 Phase

**Figure 2:**
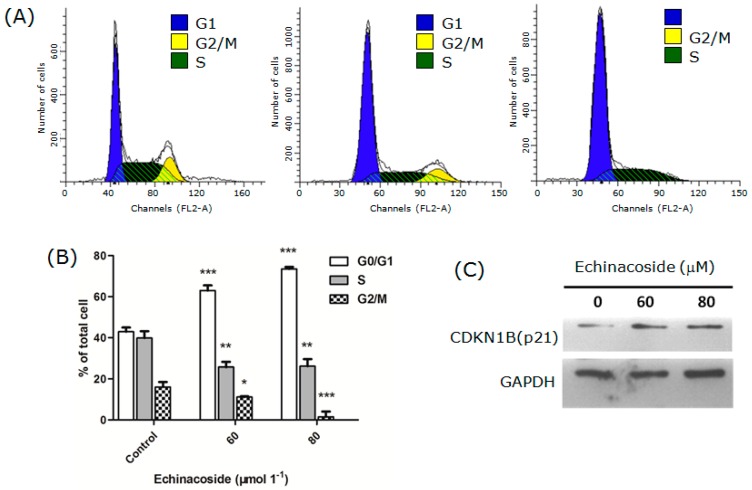
(**A**) Cell cycle analysis: Cells were treated by 0, 60 and 80 μM Echinacoside for 24 h and analyzed by the FACSCalibur flow cytometer and the ModFit software; (**B**) Quantification of cells at different stages of the cell cycle: Data from three independent experiments were analyzed by the GraphPad Prism software (* *p* < 0.05, ** *p* < 0.01 and *** *p* < 0.001 *vs*. vehicle control); and (**C**) Western blot analysis of CDKN1B (p21).

To understand how Echinacoside treatment caused the inhibition of cell proliferation, we measured the number of cells at different stages of the cell cycle by flow cytometry. The results showed that Echinacoside treatment reduced the percentage of cells in both S and G2/M phases, while the percentage of cells in G1 phase increased significantly (Figure 2A,B). After Echinacoside treatment, cells in G2/M phase decreased from 16.08% (control) to 10.88% (60 μM Echinacoside) and 2.7% (80 μM Echinacoside), and cells in S phase decreased from 41.92% (control) to 24% (60 μM Echinacoside) and 22.8% (80 μM Echinacoside) respectively (Figure 2B); while cells in G1 phase increased from 42% (control) to 65.12% (60 μM Echinacoside) and 74.5% (80 μM Echinacoside) respectively. These results showed that Echinacoside induced cell cycle arrest and blocked the cells at G1 phase.

We then analyzed the protein level of the G1/S-CDK blocker and DNA synthesis inhibitor CDKN1B (p21) by Western blot (Figure 2C). Treatment by 60 and 80 μM Echinacoside for 24 h increased the level of CDKN1B (p21), suggesting that Echinacoside blocked cell proliferation by inducing upregulation of CDKN1B (p21).

### 2.3. Echinacoside Induced Apoptosis in SW480 Cells

**Figure 3:**
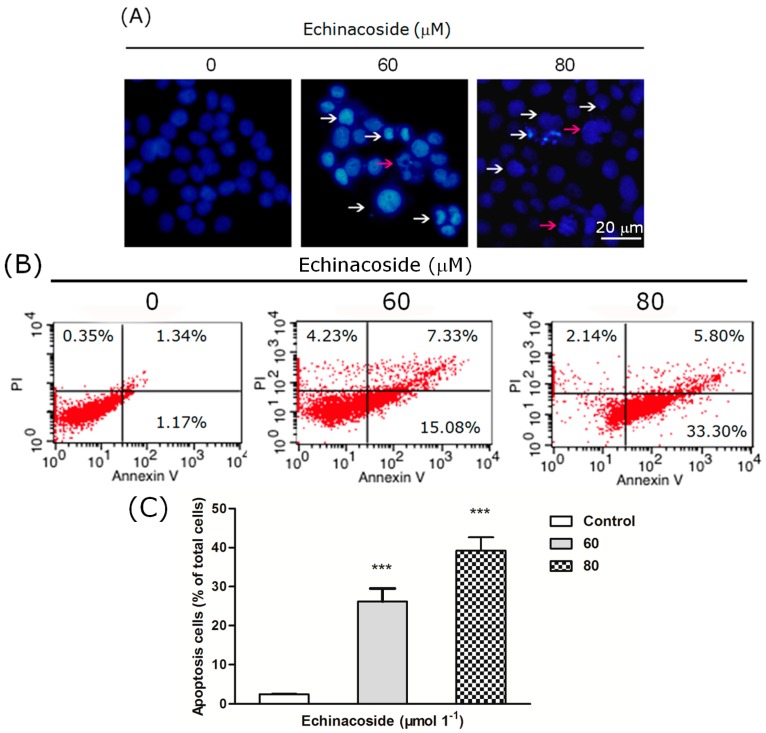
(**A**) Images of 4,6-diamidino-2-phenylindole (DAPI)-stained cells: Cells were treated by 0, 60 and 80 μM Echinacoside for 24 h, some apoptotic cells were marked by white and red arrows (scale bar = 20 μm); (**B**) Flow cytometry analysis of apoptosis: Cells were stained by Annexin V-FITC and Propidium Iodide, and analyzed by the FACSCalibur flow cytometer and the Cell Quest software; (**C**) Quantification of apoptotic cells from three independent experiments, data were analyzed using the GraphPad Prism software (*** *p* < 0.001 *vs*. vehicle control).

Another factor that may account for the inhibition of proliferation is cell death. We then examined the nuclear morphology of Echinacoside-treated SW480 cells after staining with the DNA dye DAPI. 24 h treatment by 60 and 80 μM Echinacoside increased the number of hallmarks of apoptosis, including pyknosis (shrinkage) and condensed chromatin (brighter nuclei) (Figure 3A).

To examine if Echinacoside induced apoptosis, we analyzed the cells by flow cytometry after staining with Annexin V-FITC and Propidium Iodide. Twenty four-hour treatment by Echinacoside increased the percentage of apoptotic cells from 2.5% (control) to 27% (60 μM) and 40% (80 μM), a 10- and 20-fold increase, comparing to the untreated sample (Figure 3B,C). Analysis of similarly treated SK-HEP-1 hepatoma cells showed that apoptosis was also induced in these cells by Echinacoside. The percentage of apoptotic cells after 24 h treatment by Echinacoside increased from 3.17% (control) to 12.84% (60 μM) and 22.77% (80 μM) respectively.

**Figure 4:**
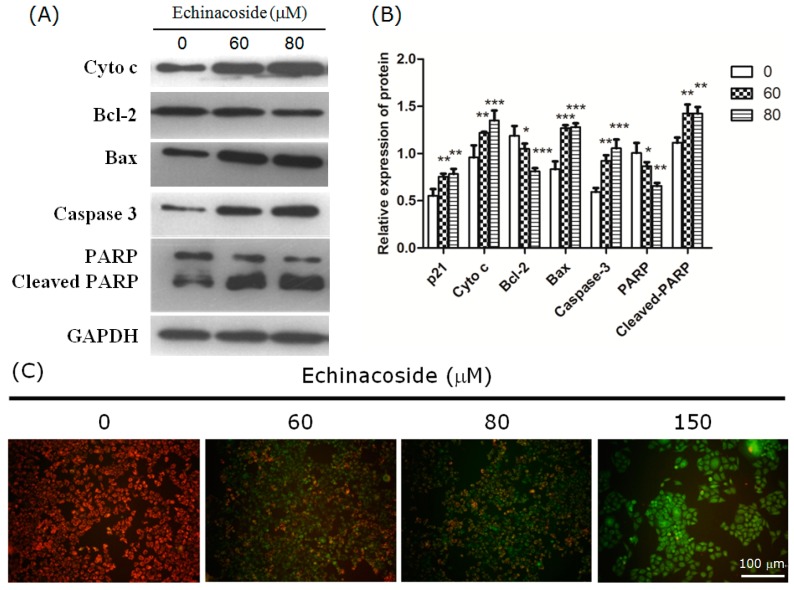
(**A**) Representative images of Western blot: Cells were incubated with 0, 60 and 80 μM Echinacoside for 24 h; (**B**) Quantification of protein level from three independent experiments by the Tanon Image Analysis software (* *p* < 0.05, ** *p* < 0.01 and *** *p* < 0.001 *vs*. vehicle control); and (**C**) Measurement of mitochondrial membrane potential: Cells were treated with different doses of Echinacoside for 24 h, and then stained with JC-1 dye. Scale bar = 100 μm.

To investigate if the apoptosis induced by Echinacoside is caspase-dependent, we analyzed caspase-dependent apoptosis-associated proteins by Western blot. The results showed that 24 h treatment by 60 and 80 μM Echinacoside clearly increased the level of active caspase 3 and cleaved PARP proteins in SW480 cells (Figure 4A,B), indicating that Echinacoside activated caspase-dependent apoptosis. The Western blot results also showed that Bax and cytochrome c levels increased significantly, while Bcl2 level decreased, suggesting that Echinacoside treatment activated the mitochondria-dependent intrinsic apoptosis pathway.

A distinctive early event of the intrinsic apoptosis pathway is the disruption of mitochondrial membrane integrity. The mitochondrial membrane potential probe JC-1 forms aggregates in the mitochondria of healthy cells, which emit red fluorescence. Loss of mitochondrial membrane potential causes JC-1 to separate into monomers, which emit green fluorescent. Staining with JC-1 showed that 24 h treatment by Echinacoside dose-dependently increased the intensity of green fluorescence, while red fluorescence decreased significantly (Figure 4C), suggesting that Echinacoside treatment caused a significant loss of mitochondrial membrane potential in SW480 cells.

### 2.4. Echinacoside Increased the Level of Oxidized Guanine 8-oxoG in SW480 Cells

**Figure 5:**
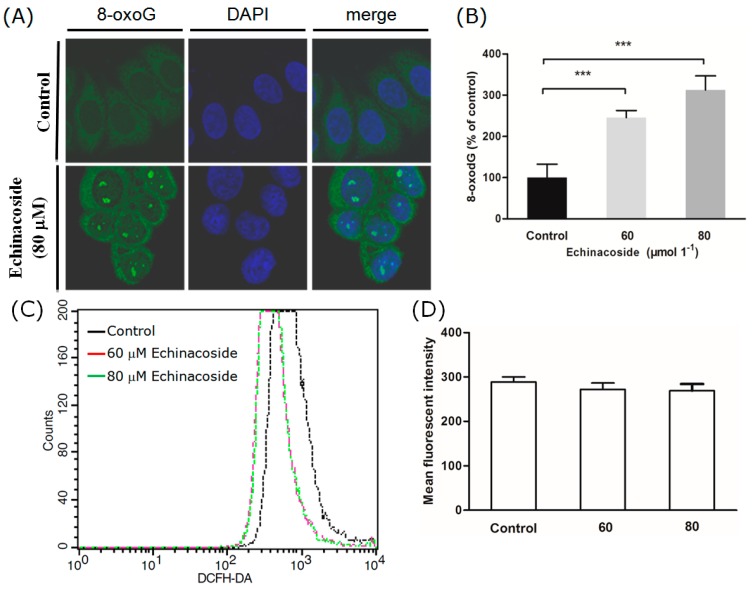

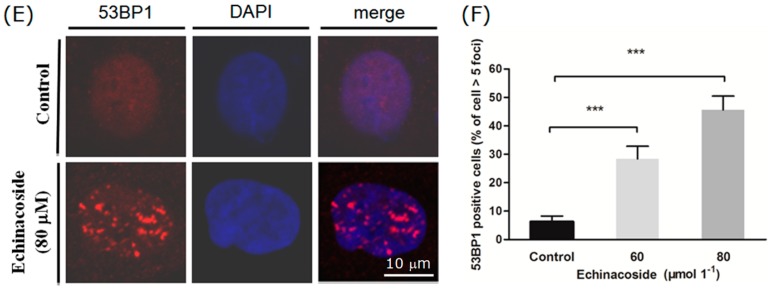
Examination of cellular 8-oxoG and 53BP1. (**A**) Representative images of cells stained by Alexa 488-conjugated avidin and DAPI: Cells were treated with Echinacoside for 24 h; (**B**) Quantification of 8-oxoG content by measuring Alexa 488-fluorescent intensity (*** *p* < 0.001 *vs*. vehicle control); (**C**) Measurement of cellular ROS by flow cytometry: Control and Echinacoside-treated cells were loaded with the ROS probe DCFH-DA, and DCF fluorescent intensity was measured by the FACSCaliber flow cytometer; (**D**) Quantification of ROS from three independent experiments; (**E**) Representative images of cells stained by anti-53BP1 antibody; Scale bar = 10 μm; (**F**) Quantification of 53BP1 positive cells: Cells with > 5 53BP1 foci were counted manually, numbers shown were average of three coverslips (*** *p* < 0.001 *vs*. vehicle control).

Although Echinacoside is best known for its anti-oxidative bioactivity, there were also studies reporting that treatment with extract of Herba *Cistanche* stimulated ATP-generating capacity and increased ROS generation in mitochondria of cardiomyocytes [24]. The cellular and mitochondrial dNTP pool is a significant target of ROS [19]. To check if Echinacoside increased oxidation of dNTPs in SW480 cancer cells, we compared the content of oxidized guanine, 8-oxoG, in SW480 cells before and after Echinacoside treatment. Avidin has been shown to bind to 8-oxoG with high specificity [25]. Staining with Alexa 488-conjugated avidin revealed that 24 h treatment by Echinacoside significantly increased intracellular 8-oxoG level in SW480 cells (Figure 5A). The effect was dose-dependent, 60 μM Echinacoside caused a 150% increase of averaged fluorescent intensity (Figure 5B); and 80 μM Echinacoside resulted in a bigger increase (330%) (Figure 5B). These results showed that Echinacoside treatment increased oxidation of nucleotide bases in the nucleotide pool and/or in DNA molecules.

To find out whether ROS was increased by Echinacoside to cause the increased oxidation of nucleotide bases, we measured intracellular ROS production by the fluorescent probe DCFH-DA. Surprisingly, 24 h treatment by both 60 and 80 μM Echinacoside induced a slight but not significant decrease in intracellular ROS content (Figure 5C,D). Forty eight-hour treatment yielded a similar result. Thus, mechanisms other than increasing ROS were likely to be responsible for the oxidative damages caused by Echinacoside.

### 2.5. Echinacoside Caused Extensive DNA Damages in SW480 Cells

8-oxoG incorporation into DNA will stimulate DNA repair processes that will generate single-strand DNA breaks (SSB) and gaps [26]. Acute increases in cellular SSB levels can saturate cellular repair capacity, leading to formation of numerous double-strand DNA breaks (DSB) [27]. 53BP1 is a protein that binds to sites of DSB and functions early in the cellular response to DSB [28]. Fluorescent immunostaining showed that treatment by 60 and 80 μM Echinacoside for 24 h induced a dramatic increase in the number of cells with strongly stained nuclear 53BP1 foci (Figure 5E,F). After treatment by 80 μM Echinacoside for 24 h, 47% of the cells contained more than five bright nuclear 53BP1 foci (Figure 5F).

Taking together, these results suggested that Echinacoside treatment caused extensive DNA damages through oxidation of nucleotide bases in SW480 cells. Sizable or acute increase of oxidized bases in DNA will saturate cellular repair capacity, leading to activation of DNA damage response signaling, such as cell cycle arrest and apoptosis [26,27,28]. Since Echinacoside treatment did not increase ROS level in SW480 cancer cells, other mechanisms, such as inhibition of nucleotide pool sanitization [29] or DNA repair [26], may be responsible for the observed effects of Echinacoside. Cancer cells, including colorectal cancer, are extremely heterogeneous at the molecular level [30,31]. Differences in expression or activity of enzymes involved in nucleotide pool sanitization or DNA mismatch- and base excision repair pathways may explain why some cell lines were insensitive to Echinacoside treatment. Different responses to aspirin have also been reported, whereby colorectal cancer patients with specific mutations in the PIK3CA gene responded to aspirin treatment, while those with a wild type PIK3CA gene did not [32,33]. SW480 cells do not harbor mutated PIK3CA gene [34], thus a connection between Echinacoside and PIK3CA mutations cannot be made here, and the mechanisms underlying Echinacoside’s apoptosis-inducing activity remain to be investigated in future studies.

## 3. Experimental Section

### 3.1. Cells and Chemicals

Human colorectal adenocarcinoma SW480 cell line was purchased from ATCC. The cells were cultured in Dulbecco’s modified Eagle’s medium (DMEM) (Gibco, Grand Island, NY, USA) containing 10% fetal bovine serum (FBS) (Gibco) and 1% (*v*/*v*) penicillin-streptomycin (Sigma-Aldrich, St. Louis, MO, USA) at 37 °C in a humidified atmosphere containing 5% CO_2_. Echinacoside was purchased from Yuanye Biotechnology, Shanghai, China (HPLC > 98%). Stock solutions were prepared in DMSO (Sigma-Aldrich), and working solutions were prepared in cell culture medium.

### 3.2. MTT Viability Assay

Cells were seeded in 96-well plates at 1 × 10^4^ cells per well for 12 h, and then incubated in the presence of Echinacoside or 0.01% DMSO for 24 or 48 h. At the end, 20 μL of MTT (3-(4,5-dimethylthiazol-2-yl)-2,5-diphenyl tetrazolium bromide) solution (5 mg/mL in PBS, pH 7.2) (Invitrogen, Carlsbad, CA, USA) was added to each well, and the plates were incubated for 4 h. After removing the medium containing MTT, 150 μL of DMSO was added to each well. The plates were incubated on a plate shaker for 10 min, and absorbance at 570 nm was measured by a BioRad 680 microplate reader (Hercules, CA, USA). Each experiment was conducted twice in triplicate. Data were analyzed using the GraphPad Prism software (San Diego, CA, USA).

### 3.3. Colony Formation Assay

Cells were seeded in 6-well plates at a density of 1 × 10^4^ per well for 12 h, and then incubated in the presence of Echinacoside or 0.01% DMSO for 10 days. The plates were washed with PBS, and cells were fixed with ice-cold methanol, and stained with crystal violet solution (Sigma-Aldrich) (0.5% in 25% methanol). Images were photographed after plates were dried overnight. The crystal violet crystals were dissolved in 70% ethanol, and absorbance at 595 nm was measured by a BioRad microplate reader. Data were analyzed using the GraphPad Prism software.

### 3.4. DNA Fragmentation Analysis with DAPI Fluorescence Staining

After drug treatment, cells in 24-well plates were washed once in PBS, fixed in ice-cold 4% paraformaldehyde (PFA) (Sigma-Aldrich) in PBS for 20 min, and then stained with 4,6-diamidino-2-phenylindole (DAPI) (Sigma-Aldrich) (0.2 μg/mL) for 10 min at room temperature. After washing with PBS, cells were sealed in VECTASHIELD Mounting Medium (Vector Laboratories, Burlingame, CA, USA). Nuclear morphology was photographed with an Olympus fluorescent microscope.

### 3.5. Cell Cycle Analysis

Cells were seeded in 96-well plates at 1 × 10^4^ cells per well for 12 h, and then incubated in the presence of Echinacoside or 0.01% DMSO for 24 h. At the end, cells were harvested with trypsin (Invitrogen), washed in PBS twice, and then fixed with ice-cold ethanol (70%) for 2 h at −20 °C. Fixed cells were washed twice in cold PBS and resuspended in 300 μL of freshly prepared PBS with 0.1% Triton X-100, 0.2 mg/mL DNase-free RNase A (Sigma), 10 μg/mL Propidium Iodide (PI) (Roche, Indianapolis, IN, USA). After incubation at 37 °C in the dark for 20 min, cells were filtered through nylon mesh (Filcon, BD Bioscience, San Jose, CA, USA), loaded to the FACSCalibur flow cytometer (BD Biosciences). Data were analyzed using the ModFit software (Topsham, ME, USA).

### 3.6. Apoptosis Analysis

Cells were seeded in 96-well plates at 1 × 10^4^ cells per well for 12 h, and then incubated in the presence of Echinacoside or 0.01% DMSO for 24 h. At the end, cells were examined using an Annexin V-FITC Apoptosis detection kit (Bestbio, Shanghai, China) according to the manufacturer’s instructions. Cells were washed twice with PBS and then resuspended in binding buffer. 5 μL each of Annexin V-FITC and Propidium Iodide (PI) (Roche) were added sequentially and the cells were incubated for 15 min in the dark at room temperature. The cells were loaded onto the FACSCalibur flow cytometer (BD Biosciences). Data were analyzed using the Cell Quest software (BD Biosciences).

### 3.7. Immunofluorescent Staining of 53BP1

SW480 cells were seeded on round coverslips in 24-well plates. After treatment with Echinacoside or 0.01% DMSO for 24 h, cells were fixed with 4% PFA for 20 min and blocked with 15% FBS (0.1% Triton X-100) for 1 h at room temperature. Fixed cells were incubated for 2 h at room temperature with rabbit anti-53BP1 antibody (Bethyl Laboratories, Montgomery, TX, USA) diluted 1:1000 in the blocking solution, followed by staining with Cy3-conjugated goat anti-rabbit secondary antibody (Jackson ImmunoResearch Laboratories, West Grove, PA, USA) diluted 1:500 in blocking buffer for 1 h at room temperature. After washing in PBS for 3 × 5 min, the coverslips were sealed on glass slides in VECTASHIELD Mounting Medium with DAPI. Images were taken by a Zeiss LCM 510 confocal microscope (Carl Zeiss, Jena, Germany).

### 3.8. 8-oxoG Incorporation Assay

Intracellular 8-oxoG was measured by staining with Alexa 488-conjugated avidin (Invitrogen) [25]. Cells were seeded on round coverslips in 12-well plates. After treatment with Echinacoside or 0.01% DMSO for 24 h, cells were fixed with ice-cold methanol for 20 min followed by incubation in Tris-buffered saline (TBS) (Fisher Scientific, Rockford, IL, USA) with 0.1% Triton X-100 (Sigma-Aldrich) for 15 min. The samples were blocked in TBS with 0.1% Triton X-100 and 15% FBS for 1 h at room temperature, and then stained with Alexa 488-conjugated avidin (0.5 μg/mL) in blocking solution for 1 h at 37 °C. After washing in PBS for 3 × 5 min, the coverslips were sealed on glass slides in VECTASHIELD Mounting Medium with DAPI (Vector Laboratories). Images were taken and analyzed by a Zeiss LCM 510 confocal microscope.

### 3.9. Western Blot Analysis

Cells grown and treated in 6-well plates were scraped in RIPA buffer (150 mM NaCl, 1.0% IGEPAL CA-630, 0.5% sodium deoxycholate, 0.1% SDS, and 50 mM Tris, 1 mM PMSF, pH 8.0) (Sigma). Total protein concentration was measured by BCA kit (Dingguo, Changchun, China) according to the manufacturer’s instructions. Samples were denatured at 95 °C for 10 min, separated on 4%–12% SDS-PAGE gel, and then transferred to PVDF membranes (Millipore, Billerica, MA, USA). Membranes were blocked in 5% fat-free milk in TBST for 2 h and incubated with primary antibodies (Bcl-2, sc-492, Santa Cruz Biotechnology, Santa Cruz, CA, USA; Bax, Santa Cruz, sc-493; cyto c, Santa Cruz, sc-7159; p21, ab109520, Abcam, Cambridge, MA, USA; caspase 3, Abcam, ab32042; PARP, Bioss, bs-2318R; 53BP1, Bethyl Laboratories, A300-272A) overnight at 4 °C. After washing with PBS, membranes were incubated with HRP-conjugated secondary antibodies (Jackson ImmunoResearch Laboratories) and visualized with ECL Western blotting detection kit (Transgen Biotech, Changchun, China). Photos were captured and analyzed by an Imager (Tanon, Shanghai, China).

### 3.10. Measurement of Cellular ROS

Intracellular ROS was measured by flow cytometry using a cell-based ROS assay kit (Beyotime Biotechnology, Haimen, China; S0033). At the end of Echinacoside treatment, the cells were washed twice with PBS, and incubated with 10 μM dichlorofluorescin diacetate (DCFH-DA) for 30 min at 37 °C. The cells were then trypsinized and analyzed by the FACSCaliber flow cytometer (BD Biosciences). Intracellular ROS levels were expressed as the average DCF fluorescence intensity of the cells.

### 3.11. Measurement of Mitochondrial Membrane Potential

Mitochondrial membrane potential was measured using the JC-1 dye (Beyotime Biotechnology, C2006) following manufacturer’s instructions. Control and Echinacoside-treated cells were incubated with JC-1 for 20 min and then examined with an Olympus fluorescent microscope.

### 3.12. Statistical Analysis

Statistical analysis was performed using the GraphPad Prism Software. Significance was calculated using one-way analysis of variance (ANOVA) and *p* < 0.05 was considered statistically significant. Results were expressed as mean ± SD.

## 4. Conclusions

In this study, we tested the effects of Echinacoside on a variety of human cancer cell lines. Surprisingly, we found that Echinacoside inhibited proliferation of human SK-HEP-1 hepatoma, MCF-7 breast cancer and SW480 colorectal cancer cells. Further analyses found that Echinacoside arrested SW480 cells at G1 phase, and induced caspase-dependent apoptosis by activating the mitochondria-associated intrinsic apoptosis pathway. Cell cycle arrest was related to upregulation of the G1/S-CDK blocker and DNA synthesis inhibitor CDKN1B (p21), and apoptosis was associated with downregulation of the anti-apoptotic protein Bcl-2 and upregulation of pro-apoptotic Bax, as well as loss of mitochondrial membrane potential. The significant increase of intracellular oxidized form of guanine, 8-oxoG, suggests that there were increased oxidization of dNTPs and/or DNA molecules, which may have caused the cell cycle arrest and apoptosis in SW480 cancer cells. While the mechanisms underlying Echinacoside’s effects on oxidative damage and apoptosis remain to be characterized, these results support Echinacoside as a novel chemical scaffold for the development of anticancer drugs.
